# Bacteriophages: A Challenge for Antimicrobial Therapy

**DOI:** 10.3390/microorganisms13010100

**Published:** 2025-01-07

**Authors:** Nallelyt Segundo-Arizmendi, Dafne Arellano-Maciel, Abraham Rivera-Ramírez, Adán Manuel Piña-González, Gamaliel López-Leal, Efren Hernández-Baltazar

**Affiliations:** 1 Laboratorio de Microbiología y Parasitología, Facultad de Farmacia de la, Universidad Autónoma del Estado de Morelos, Cuernavaca 62209, Mexico; san_ff@uaem.mx; 2 Laboratorio de Biología Computacional y Virómica Integrativa, Centro de Investigación en Dinámica Celular, Universidad Autónoma del Estado de Morelos, Cuernavaca 62209, Mexico; dafneunadm@nube.unadmexico.mx (D.A.-M.); apinag1700@alumno.ipn.mx (A.M.P.-G.); 3 Laboratorio de Estudios Ecogenómicos, Centro de Investigación en Biotecnología, Universidad Autónoma del Estado de Morelos, Cuernavaca 62209, Mexico; abraham.rivera@uaem.mx; 4 Laboratorio 1 de Tecnología Farmacéutica, Facultad de Farmacia de la, Universidad Autónoma del Estado de Morelos, Cuernavaca 62209, Mexico

**Keywords:** phage therapy, multidrug-resistant bacteria (MDR), antimicrobial resistance, phage-based therapeutics, CRISPR, phage stability, biofilm inhibition, synthetic biology

## Abstract

Phage therapy, which involves the use of bacteriophages (phages) to combat bacterial infections, is emerging as a promising approach to address the escalating threat posed by multidrug-resistant (MDR) bacteria. This brief review examines the historical background and recent advancements in phage research, focusing on their genomics, interactions with host bacteria, and progress in medical and biotechnological applications. Additionally, we expose key aspects of the mechanisms of action, and therapeutic uses of phage considerations in treating MDR bacterial infections are discussed, particularly in the context of infections related to virus–bacteria interactions.

## 1. Introduction

**Figure 1:**
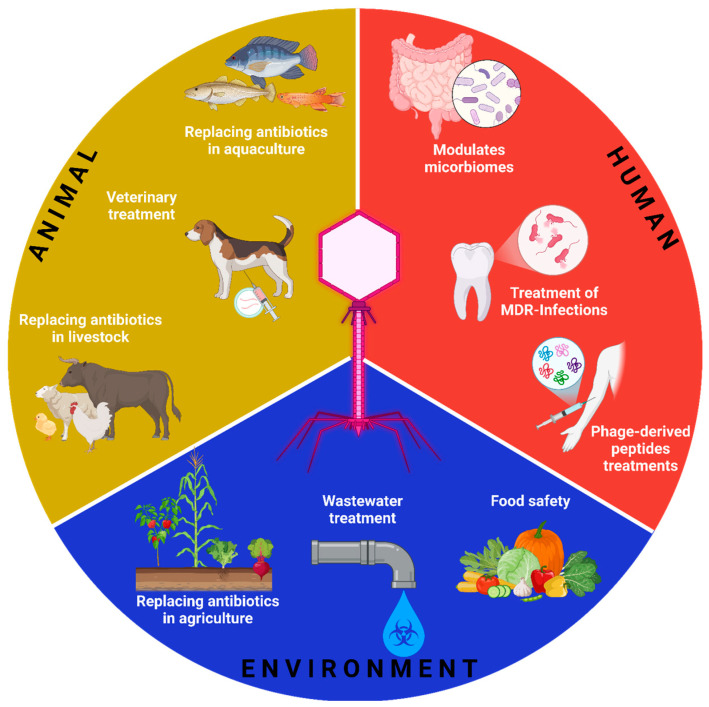
Applications of bacteriophages in different environments. Bacteriophages are used in multiple areas, from food safety and wastewater treatment to the replacement of antibiotics in agriculture, aquaculture, and livestock to the modulation of microbiomes to the treatment of infections by multidrug-resistant bacteria and the development of treatments based on phage-derived peptides. Created in https://Biorender.com. (https://app.biorender.com/illustrations/674a2864ec7228acf3cce191, accessed on 10 December 2024).

Viruses are the most diverse and abundant organisms in the biosphere [1]. Within the world of viruses, bacteriophages are those viruses that only infect bacteria. Certainly, the history of bacteriophages is quite fascinating and spans over a century [2]. Bacteriophages were discovered independently by two scientists in the early 20th century: Frederick Twort in 1915 and Félix d’Hérelle in 1917 [3]. Félix d’Hérelle, coined the term “bacteriophage” and concluded that he had discovered bacterial viruses. In recent years, massive sequencing technologies, together with new bioinformatics tools, have made it possible to identify unculturable phage populations [4], among which crassphage [5] and gubaphage [6] stand out. Both discovered through metagenomic analysis of human intestinal microbiomes have proven to be among the most abundant groups of bacteriophages in this environment, although their biology remains unknown. These phages play a key role in the regulation of intestinal bacterial communities and can have a significant impact on human health by influencing the composition of the microbiota (Figure 1). Phages have recently gained importance owing to the global antibiotic resistance crisis [7]. In the sense of using them as an alternative to antibiotics to combat superbugs [8], this article provides a brief review of the relevant aspects of phages in the last decades. Finally, we consider that there are still significant challenges to using phages as antimicrobial elements, so it is important to continue investigating since antimicrobial resistance is a public health problem that affects the entire world.

## 2. Phage Classification

**Figure 2:**
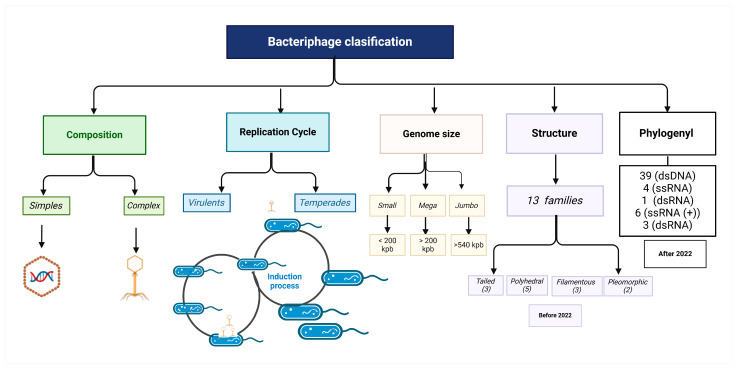
Characteristics to bacteriophage classification. Brackets indicate the number of families of each group of bacteriophages. Created in https://Biorender.com. (https://app.biorender.com/illustrations/6747f724c29262265170d394?slideId=6a206afd-ad7c-4a61-986b-e2d09a8a067f, accessed on 12 December 2024).

After the discovery of bacteriophages, serological cross-reactions and host range became some of the primary criteria for their classification [9,10,11]. Later, in the 1950s, the advent of electron microscopy provided detailed visualizations of phage structures, leading to a classification system based on their morphology [12]. This approach allows researchers to categorize bacteriophages according to features such as the presence of tails, capsids, and overall shapes, such as icosahedral or filamentous structures (anexo) (Table S1). In April 2022, the International Committee on Taxonomy of Viruses (ICTV) Bacterial Viruses Subcommittee brought together forty-five experts in virology, bioinformatics, evolution, and structural biology in Oxford, United Kingdom, to reach a consensus on virus classification methodologies (Figure 2) [13,14,15,16]. The goal was to develop an integrated and consistent taxonomic framework. Central to their discussions was how to construct evolutionary taxonomy for viruses.

**Figure 3:**
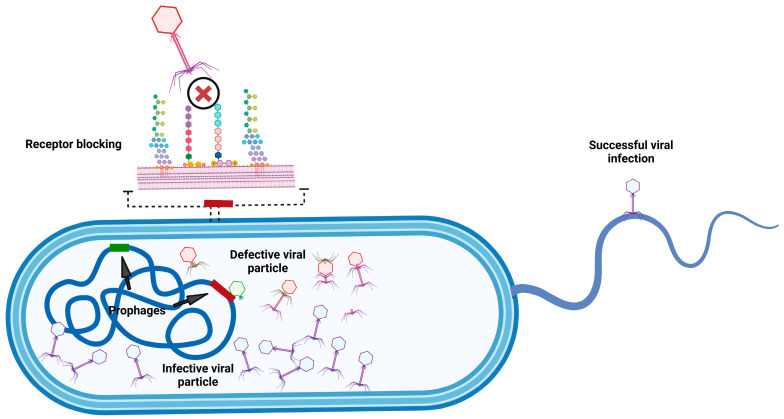
Potential functions of orthologous prophages conserved in their hosts. Phages that use pili for host infection have certain advantages over those that rely on specific receptors. Degraded prophages can interfere with the assembly of other phages (shown in red and green), resulting in the formation of defective particles. Some prophages (yellow) can block or modify the receptor to prevent superinfection. Created in https://Biorender.com (https://app.biorender.com/illustrations/674b8d7e17e4553b2d92d185?slideId=e3bb9a60-5d25-424c-9f11-1e39e2d4a470, accessed on 10 December 2024).

The recent consensus on virus taxonomy offers a detailed and methodical approach for classifying viruses based on genomic sequence comparison and phylogenetic analysis, emphasizing evolutionary relationships as the foundation for taxonomic assignments. The insistence that virus taxonomy is rooted in evolutionary history is logical and scientifically sound. By ensuring that taxonomic groups are monophyletic, virologists aim to create a system in which viruses within a group share a common ancestor, thereby accurately reflecting their natural history and evolutionary relationships [17]. The ICTV establishes specific thresholds for pairwise nucleotide sequence similarity and orthologous gene sharing, which determine the classification hierarchy (family, genus, species, etc.) for each phage ranked within the taxonomy. Two phages are classified under the same species if their genomes are more than 95% identical at the nucleotide level across their entire genome length. In its effort to establish cohesive, distinct, reproducible, and monophyletic genera, the subcommittee has set a 70% nucleotide identity across the full genome length as the threshold for defining genera. At the family level, there have been no fixed demarcation criteria in the past. However, it has been proposed that the family be represented by a cohesive and monophyletic group in the main proteome-based clustering and that members of the family share a significant number of orthologous genes. To achieve this, several bioinformatic tools have emerged, such as VIRIDIC [17] for genus and species determination. Tools like VirClust [18] and vContact2 [19] have been developed for family-level classification to enhance the analysis of viral genomes and phylogeny. These approaches enable the identification of conserved genetic markers such as core genes, average amino acid identity, and nucleotide alignment [19,20]. This is used to accurately define viral clades, ensuring that taxonomic classifications align with the evolutionary data [13]. In this sense, the current genome-based taxonomy of the order *Crassvirales* includes four families (*Intestiviridae*, *Crevaviridae*, *Suoliviridae*, and *Steigviridae*), 11 subfamilies, 42 genera, and 73 species [21]. Moreover, the gubaphage clade does not yet have an official classification within the International Committee on Taxonomy of Viruses (ICTV) system. Although great progress and efforts have been made by the ICTV, phages present unique challenges in evolutionary taxonomy because of their highly dynamic genomes. Phages are known for their ability to engage in horizontal gene transfer and recombination with bacterial hosts and other phages. For example, Figure 3 shows how the presence of orthologous prophages in hosts could recombine with virulent phages to produce defective viral particles. However, these recombination events can also produce new and diverse infective viral particles [21,22,23,24]. This genetic feature can obscure phylogenetic relationships and complicate the efforts to create monophyletic taxonomic groupings [25]. Despite these challenges, evolutionary taxonomy remains essential. Phylogenetic analysis allows for the identification of evolutionary lineages and sheds light on the evolutionary dynamics between phages and their bacterial hosts, which is critical for understanding the ecological and evolutionary roles of phages. Therefore, the correct characterization of phages is important if the scientific community wants to use them as an alternative tool to antibiotics in the sense that when we identify phage species, we may also be able to identify their potential hosts.

### 2.1. Mechanisms of Adsorption and Infection

**Figure 4:**
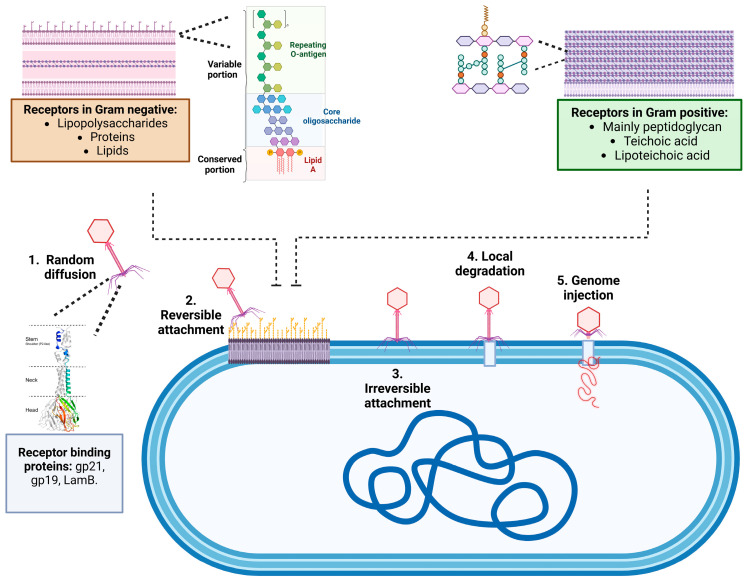
Phage adsorption process. Phage adsorption to their host occurs through the interaction between receptor-binding proteins (RBPs) at the tip of the phage tail and receptors on the bacterial cell envelope. This process unfolds in three stages: (1) random diffusion through the medium, (2) reversible attachment to the bacterial surface, and (3) irreversible attachment. Depolymerases may facilitate adsorption by cleaving capsular polysaccharides that otherwise obstruct receptor access. After attachment, virion-associated lysins may locally degrade the peptidoglycan (PG) layer (4), and finally, conformational changes in the phage structure trigger the injection of the genome into the host (5). Created in https://BioRender.com. (https://app.biorender.com/illustrations/6749ff7c4452d3ac3336507e?slideId=c8c84890-7a16-40b5-a20a-0414b025d1ae, accessed on 15 December 2024).

The process of phage particle adsorption to a bacterium triggers a series of molecular mechanisms of vital importance, such as tail contraction and genetic material ejection from the phage, which are crucial for the infective process of the host bacterium. This process is finely regulated by the proteins of the bacteriophage adsorption apparatus named receptor-binding proteins (RBPs) [25]. The process consists of three stages: initial contact, reversible binding, and irreversible anchoring. In the first stage of initial contact, the bacteriophage approaches the surface of the bacterial cell. This contact is generally driven by physical and stochastic factors, such as Brownian motion, which causes the phage particles to move and collide randomly with the bacterium. At this stage, the interaction is weak and non-specific. Once the bacteriophage is close to the bacterial cell, it begins to interact more specifically with the surface of the cell. This involves interactions between viral proteins (usually on the tail fibers or spicules of the phage) and specific receptors on the bacterial cell surface (such as proteins, carbohydrates, or lipopolysaccharides). These interactions are reversible, meaning the phage can detach from the cell at this stage if the interaction is not strong enough. After reversible binding, the bacteriophage undergoes conformational changes that lead to a stronger and more stable attachment to the bacterial surface. This step often involves more specific binding to receptors or structural components of the bacterial cell wall. Once irreversible binding is achieved, the phage can inject its genetic material into the bacterial cell to initiate the infection cycle as shown in Figure 4 [24,25,26,27].

This can vary depending on whether the bacterium is Gram-negative (featuring lipopolysaccharides and outer membrane components) or Gram-positive (characterized by peptidoglycan layers, embedded teichoic acids, lipoteichoic acids, and associated proteins). There are various receptors in the cell wall of Gram-negative bacteria that interact with the receptor-binding proteins (RBPs) of bacteriophages, as shown in Table 1. These receptors include cell wall proteins, polysaccharides, and structures such as flagella, pili, and capsules (see Figure 4). In Gram-negative bacteria, the peptidoglycan layer is thin and located beneath the outer membrane, which consists of a lipid bilayer with proteins, polysaccharides, and lipids. Lipopolysaccharides (LPS) are complex structures that include lipid A, the core polysaccharide, and the O-polysaccharide (O-antigen). Bacteria containing all three LPS components are referred to as smooth (S) type, while those lacking the O-polysaccharide are considered rough (R) type [28]. Phages that are specific to S-type strains tend to target the O-polysaccharide, limiting their host range, while phages that can bind to R-type cells have a broader host range. Phages can also degrade the polysaccharides in the O-antigen structure through enzymes, especially those from the *Podoviridae* family. In addition, other bacterial structures such as flagella, pili, and capsules serve as phage receptors. For example, phages can reversibly bind to flagella, but irreversible adsorption occurs when the phages attach to receptors on the cell wall. Pili, structures involved in bacterial conjugation, also serve as specific receptors for certain phages. Capsules, composed of polysaccharides or proteins, allow for phage adsorption through enzymatic hydrolysis of the exopolysaccharides since they contain virion-associated enzymes called polysaccharide depolymerases and lysins, which recognize, bind to, and degrade polysaccharide compounds followed by irreversible binding to the receptors on the cell wall. Bacteriophage-encoded depolymerases can be categorized into two primary classes based on their mode of action: hydrolases (EC 3) and lyases (EC 4). Both classes facilitate the cleavage of polysaccharides into soluble oligosaccharides, thereby breaking down the carbohydrate barrier. The depolymerases responsible for the degradation of CPS, EPS, or O-polysaccharide can be virion associated as an integral part of the virion particle or can be in a soluble form, being released during bacterial cell lysis without being integrated in a phage particle [10,29,30].

Virion-associated lysins (VALs) are a type of lytic transglycosylase, distinct from hydrolases. While the phage tail is the most common site for VAL attachment, it is not the only location. VALs can also be anchored to the phage neck or baseplate or integrated into the viral membrane. The specificity of VALs is categorized into three types: (i) glycosidases, which are further divided into lysozymes, glucosaminidases, and lytic transglycosylases, all of which cleave one of the two glycosidic bonds in the glycan chain; (ii) amidases, which break the amide bond between the lactyl group of N-acetylmuramic acid and L-alanine in the stem peptide; and (iii) endopeptidases, which cleave within the stem peptide or its cross-link [31,32].

Peptidoglycan, or murein, is a key component of the cell wall in Gram-positive bacteria and plays an important role in bacteriophage adsorption (see Figure 3). This molecule is a polymer composed of units of amino acids and sugar derivatives like N-acetylglucosamine and N-acetylmuramic acid, which are connected by glycosidic bonds to form glycan tetrapeptide sheets. In Gram-positive bacteria, the cell wall is thicker due to a higher number of peptide cross-links that bind the peptidoglycan chains together. Another relevant component in phage adsorption is teichoic acid, a polysaccharide made of glycerol phosphate or ribitol phosphate and amino acids, which is linked to the muramic acid of peptidoglycan. When these acids are attached to lipids in the plasma membrane, they are called lipoteichoic acids (LTA). However, few specific phage receptors in Gram-positive bacteria have been identified due to the complexity of their cell walls, which contain various components that can interfere with phage adsorption. Additionally, there is a smaller body of research on phages of Gram-positive bacteria compared to those of Gram-negative bacteria [30,31,32,33]. Few protein receptors have been identified in Gram-positive bacteria. The *Bacillus anthracis* phage γ interacts with a surface protein, whereas *B. subtilis* phage SPP1, some *Enterococcus faecalis* phages, and a few lactococcal phages bind to membrane proteins that possess a large segment exposed at the cell surface [34,35]

Out of the 30 reported phages that target Gram-positive bacteria, only 10 interact with structures other than peptidoglycan or teichoic acid. Of these, nine show interactions with teichoic acid or peptidoglycan residues for reversible binding. This highlights the importance of these components in phage adsorption to Gram-positive bacteria, particularly in important pathogens like *Bacillus*, *Staphylococcus*, *Streptococcus*, and *Corynebacterium* [34,35].

Additionally, mutations or changes in receptors on the bacterial surface may affect bacterial susceptibility to phages [36].

**Table 1:** Phage–receptor interactions by RBP and bacterial type.

Phage	Receptor-Binding Protein (RBP)	Host Receptor	Bacterial Species	Gram Type	References
Phage T4	Hoc, J, and K proteins	Lipopolysaccharides (LPS)	*E. coli*	Gram-negative	[37]
Phage λ	LamB (Maltose porin)	Maltose/maltodextrin transporters	*E. coli*	Gram-negative	[38]
Phage DMS3	Pilus-binding protein	Type IV pili	*Pseudomonas aeruginosa*	Gram-negative	[39]
Phage MS2	Coat protein (F-pilus recognition)	F-pili (Fertility pilus)	*E. coli*	Gram-negative	[40]
Phage TLS	Receptor-binding protein	TolC	*E. coli*	Gram-negative	[41]
Phage PM2	P10 protein	Sugar moieties on the cell surface	*Pseudoalteromonas*	Gram-negative	[42]
Phage Ω8	Receptor-binding protein	Polysaccharide, outer membrane proteins	*E. coli*	Gram-negative	[43]
Phage S16	Tail fibers	Outer membrane protein (OmpC)	*Salmonella*	Gram-negative	[44]
Phage ϕ29	gp12	Teichoic acids, cell wall peptidoglycan	*Bacillus subtilis*	Gram-positive	[45]
Phage SPP1	Tailspike protein	Teichoic acid	*Bacillus subtilis*	Gram-positive	[46]
Phage Bam35	Coat proteins	N-acetyl-muramic acid (MurNAc) of peptidoglycan in the cell wall	*Bacillus thuringiensis*	Gram-positive	[47]
Phage φLC3	Receptor-binding protein	Cell wall polysaccharides	*Lactococcus lactis*	Gram-positive	[48]
Phage A511	Tailspike protein	Peptidoglycan	*Listeria monocytogenes*	Gram-positive	[49]
Phage φ812	Tail protein	Anionic backbone of wall teichoic acid	*Staphylococcus* *aureus*	Gram-positive	[50]
Phage φSLT	Phage tail tip	Poly(glycerophosphate) moiety of lipoteichoic acid (LTA)	*Staphylococcus* *aureus*	Gram-positive	[51]
Phage T3	Tailspike protein	Teichoic acid	*Corynebacterium glutamicum*	Gram-positive	[50]

### 2.2. Life Cycle of Bacteriophages

Historically, the life cycle of bacteriophages has been characterized by two primary pathways: the lysogenic cycle, also referred to as the temperate cycle, and lytic cycle [52,53]. During the lytic cycle, the phage attaches to specific receptors on the surface of the bacterium, which permeabilizes the membrane and then injects its genetic material into the cytoplasm of the host cell. Subsequently, the phage uses bacterial cellular machinery to replicate its DNA or RNA, transcribe, and synthesize viral proteins that are assembled into new phage particles within the cell. Finally, the phages lyse the infected cells, releasing new phages into the medium to infect the new bacteria [54,55]. In the lysogenic cycle, the phage’s genetic material is integrated into the bacterial genome and remains dormant as a prophage [56]. Recently, it was reported that phages in the form of prophages play a significant role in altering the genetic repertoire of their hosts by carrying in their genome virulence genes [23,56] or genes that confer resistance to antibiotics. In this sense, it has been observed that pathogenic bacteria tend to harbor more prophages [55,56,57]. Conversely, some phages, such as those from the *Inoviridae* family (filamentous phages), utilize nonlytic mechanisms to release viral particles. Rather than destroying the host cell, these phages are gradually expelled through the cell membrane via a process known as extrusion, in which viral particles assemble on the cell surface without inducing lysis [52,53,54,55,56,57,58,59]. This allows the bacterial cells to remain alive and produce phages for an extended period [60]. Several phages can employ mechanisms like a few eukaryotic viruses [61]. This mechanism involves viral particles emerging from the cell through its membrane without the lysis of the bacterial cell. One example of this type of phage is the bacteriophage M13 [60].

## 3. Structure and Organization of Bacteriophage Genomes

Bacteriophages are a genomically diverse group of viruses, and this viral diversity is probably because of polyphyletic origin. Bacteriophage genomes can exhibit widespread mosaicism owing to prophage recombination events [62]. There can be several scenarios: (1) recombination between virulent phages and prophages (harbored in the target host), (2) bacterial genomic rearrangements giving rise to new prophages and their activation, and (3) the formation of viral rearrangements during the phage replication process. Ultimately, phage genomes are made up of different combinations of modules (e.g., lysogeny module, structural protein), each with its origin. Of course, all bacteriophages contain in their genome the proteins necessary to form their progeny, which depends on their identity, and can be structural, replication, and bacterial lysis proteins.

### 3.1. Key Genes for the Replication and Assembly of Phages

**Figure 5:**
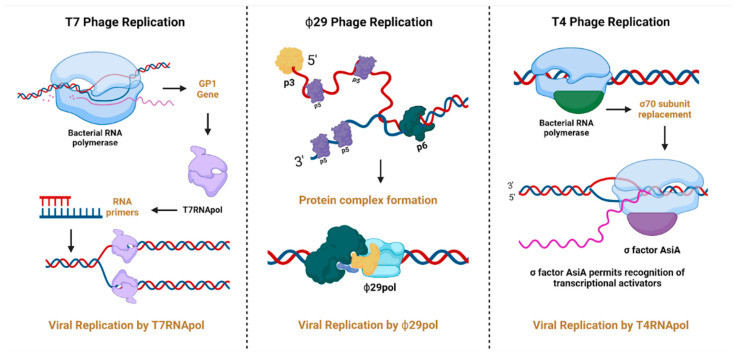
Replication mechanisms highlighting the role of DNA polymerases in bacteriophages T7, T4, and ϕ29. Created in https://BioRender.com. (https://app.biorender.com/illustrations/67410661b7b47ecc72de04e2?slideId=276310af-2041-4a16-9d28-b84be0610664, accessed on 20 December 2024).

DNA polymerases are essential enzymes for synthesizing DNA and play key roles in genome replication, DNA repair, and genetic recombination in cellular organisms and viruses, including bacteriophages. These enzymes are categorized into several families (A, B, C, D, X, Y, and RT) according to their structure and function [63,64]. However, DNA polymerases found in bacteriophages differ significantly from other known enzymes capable of catalyzing DNA synthesis [65]. These differences are both structural and functional and arise from the extensive genetic diversity of bacteriophages and the unique characteristics of their enzymes, which likely evolve under specific conditions. This selection process has led to the development of unusual properties vital for the survival of these viruses [65]. Three well-studied examples of phage replication mechanisms involving these enzymes are known: T7 DNA polymerase from family A and DNA polymerases from phages ϕ29 and T4, which belong to the B family. Despite their structural similarities, they also exhibit functional differences. The replication of the T7 bacteriophage genome occurs through a replication mechanism mediated by the host’s RNA polymerase, which transcribes the early phage genes, including the gp1 gene, which encodes the bacteriophage’s RNA polymerase. This enzyme then synthesizes RNA primers on the leading strand, which serve as starting sites for replication by the T7 DNA polymerase [66,67,68]. In contrast, ϕ29 polymerase does not require the presence of RNA polymerase. In this case, replication is initiated by the terminal protein p3, which is covalently attached to the 5′ end of DNA. Subsequently, the DNA ends become destabilized owing to the interaction with the p6 protein. Replication is initiated through the formation of a multiprotein complex involving the proteins p2, p3, p6, and ϕ29 polymerase, which synthesizes the first six nucleotides. The polymerase then dissociates from the complex and continues replication independently [68,69]. The replication process conducted by T4 DNA polymerase is even more complex and involves interactions with a larger number of protein factors. To initiate replication, proteins encoded by phage genes are required, which means that transcription occurs first. Once the necessary proteins are synthesized, replication begins at several origins (oriA, oriC, oriE, oriF, oriG) [70,71]. The choice of origin influences the requirements for specific transcriptional activators: MotA is required for oriA, oriF, and oriG, while DbpC is essential for oriE. To kick off the replication process, the host RNA polymerase must recognize the replication origins. This recognition occurs after the standard σ70 subunit is replaced by the phage-encoded σ factor AsiA. This replacement promotes the formation of an R-loop structure in the DNA strand running from 3′ to 5, which serves as a primer for the unidirectional synthesis of the leading strand. The R-loop is critical because it enables the replication machinery to move efficiently along the phage DNA [72,73,74,75]. See Figure 5.

### 3.2. Viral Versatility: How Bacteriophages Exploit Host Defenses and Drive Evolutionary Change

As mentioned above, phages lack the necessary machinery and mechanisms to replicate. Instead, they use specialized mechanisms to take over the host cell’s protein synthesis pathways to produce the viral proteins necessary to assemble new virions [76]. In this sense, bacteria and bacteriophages engage in an ongoing evolutionary arms race, where selective pressures drive reciprocal adaptations, therefore promoting genetic diversity and driving the evolution of complex biological systems, such as those involved in the development of immunity and resistance [77].

One of the primary bacterial defense mechanisms against phage infection is the adaptive immune CRISPR-Cas system. Briefly, this system takes a small fragment of the viral DNA and integrates it into its CRISPR locus, which is composed of repeat-spacer arrays (Clustered Regularly Interspaced Short Palindromic Repeats). These foreign sequence fragments are then transcribed into crRNA and tracrRNA [78], enabling the early recognition of these sequences in case of future infection. The crRNA (CRISPR RNA) and tracrRNA (trans-activating CRISPR RNA) play essential roles in CRISPR-Cas systems by guiding the Cas9 nuclease to specific target DNA sequences. The crRNA contains a spacer sequence complementary to the target DNA, enabling precise recognition, while the tracrRNA binds to both the crRNA and Cas9, forming a functional ribonucleoprotein complex. Together, these RNAs direct Cas9 activity, allowing the cleavage of the target DNA at the specified location. However, phages can resist Cas-mediated cleavage through extensive DNA modifications, such as cytosine glycosylation, which hinders mutagenesis efficiency [79]. On the other hand, prophages tend to become inactivated quickly, although their genes remain under purifying selection [80]. In other words, certain phage genes tend to eliminate deleterious mutations that may compromise functionality and retain useful functions, such as those related to structure and lysis. This pattern suggests that bacteria have “domesticated” these elements to gain adaptive advantages, including defense against other phages and the transfer of beneficial genes (Figure 5). However, once these functions are replaced by other genetic elements, prophages tend to be eliminated from the bacterial genome. One notable aspect is that domesticated phages (also called “cryptic prophages”) can provide immunity against new infections by other phages, as phage DNA integrated into the bacterial genome often blocks infection by other phages using the same receptor [81].

On the other hand, some bacteriophages encode a variety of anti-CRISPR (Acr) proteins that inhibit CRISPR-Cas immunity in their bacterial hosts. Acr proteins typically inhibit CRISPR-Cas activity by binding to and inactivating target Cas proteins [82]. Since then, the application of new computational methods has led to the discovery and characterization of over 40 families of Acr proteins [82]. These proteins possess tremendous potential as biotechnological tools. For example, a study published in 2023 explored engineered anti-CRISPR proteins, proposing their use as “off-switch” regulators in editing technologies based on CRISPR-Cas9. Other research suggests that Acr proteins exhibit a broad range of functional capabilities, which may aid in developing therapeutic tools for various diseases caused by multidrug-resistant bacteria as well as for disorders associated with defective genes, such as Alzheimer’s disease [83]. Understanding how phages adapt their anti-CRISPR strategies to counteract evolving bacterial defenses provides valuable insights into microbial ecology and is crucial for developing safer and more efficient phage therapies and other phage-derived technologies. While researchers have characterized the mechanisms of action of several of these proteins, a deeper understanding of their roles in phage infection biology is still emerging [84].

On the other hand, it was also found that phage infection can trigger reverse transcription to assemble a toxic repetitive gene from a non-coding RNA [85]. This defense mechanism employs stress signals associated with phage infection as a trigger for Defense-associated reverse transcriptases (RTs) binding to noncoding RNA templates encoded by the bacterial genome [86]. These RTs synthesize a never-ending gene containing multiple repeats, with in-frame open reading frames (ORFs) based on the noncoding RNA template. These repetitive proteins are toxic since they conduct rapid growth, which induces a cell-cycle-arrest response, and result in either dormancy or cell death [84,85,86,87,88]. Therefore, preventing the phage from completing its life cycle and the subsequent infection of nearby bacteria is important.

### 3.3. Genetic Modification of Bacteriophages

The growing global threat of multidrug-resistant bacteria has rendered many antibiotics ineffective [87]. This worldwide health problem has brought bacteriophages back into the spotlight as a viable alternative to target bacterial strains that no longer respond to conventional treatment [88].

While phage therapy is a promising strategy to fight “super-bugs” in the post-antibiotic era, its effectiveness is still limited by many challenges [89]. The most significant limitations that affect the widespread use of phage therapy are the specificity that exists between the phage and its host (bacteriophages have a notoriously narrow host range), the ability of bacteria to gain resistance against bacteriophage infections, the unavailability of suitable lytic phages for therapy, and ineffective administration methods, in some cases [89,90,91]. However, the specificity of phages facilitates the use of phages to combat multidrug-resistant bacteria due to obeying the principles of “one health,” which seeks to establish more targeted measures without impacting other microorganisms.

However, techniques such as CRISPR-based genome editing, and other synthetic biology approaches have expanded the potential to tailor bacteriophages with unprecedented precision and specificity [91]. The use of enhanced synthetic mutants to target antibiotic-resistant bacteria has already proven to be an effective way of increasing the efficacy of phage therapy overall [92]. One example would be in one case study where recombinant T4-like phages were successfully utilized to inhibit planktonic pathogenic *Escherichia. coli* by preventing biofilm formation [92]. Recombinant phages are not only helpful in combating multidrug-resistant bacteria but are also used as reporter phages for the identification of key cells. For example, for mycobacteriophage D29 [92,93], which was genetically modified using BRED (Bacteriophage Recombineering of Electroporated DNA), a technology introduces recombinant DNA into bacteriophages using electroporation to insert mutant DNA fragments into phage-infected bacterial cells for the detection of mycobacterial cells.

On the other hand, phage’s biomolecules have become indispensable in modern biology, not only against multidrug-resistant bacteria but also by contributing to the development of evolutionary tools and facilitating a wide range of advanced molecular techniques. Phage-derived peptides can be used as therapeutic agents, and these therapies offer a lower-cost production, superior stability, and low immunogenicity. Approaches such as phage display, a technology that was first described in 1985 [94], takes advantage of filamentous bacteriophages capacity to display fusion proteins after the insertion of foreign DNA fragments into the phage; in the 90s it was further developed to produce phages that express antibodies on their surfaces [95,96,97,98]. Recent advancements in phage display have also made it a valuable tool in cancer therapy by developing phage-derived peptides that when conjugated to imaging agents like radioisotopes, fluorescence markers, or nanoparticles, can improve the sensitivity and specificity of molecular imaging techniques such as PET, SPECT, MRI, and fluorescence imaging [34]. For example, the M13-derived peptide targeting human gastric mucin (MUC5AC) [97] and other phage-derived peptides like VRPMPLQ [97], which were conjugated with fluorescein to enhance contrast in dysplastic colon tissues. Another peptide, TCP-1, successfully detected dysplastic lesions in early colorectal cancer and IBD, demonstrating rapid, high-contrast imaging [98].

Additionally, the peptide QPIHPNNM was isolated through phage display and shown to bind specifically to dysplastic colonic adenomas, enhancing detection capabilities during endoscopic procedures [99,100], and peptides targeting MUC1 (which are often overexpressed in colorectal cancer) have been selected for their ability to bind dysplastic cells. These results provide a potential avenue for targeted imaging and therapy. However, challenges regarding the stability of these peptides in larger applications remain, and further research will be necessary to optimize their clinical utility.

## 4. Therapeutic Applications

Since their discovery in 1917 by Félix D’Herelle, bacteriophages have been identified as a therapy for the treatment of bacterial infections. However, the little knowledge of their biology, the appearance of antibiotics, and even the historical moments and social conditions of researchers contributed to the decline of phage therapy and the massive use of antibiotics, which meant that only 15 years after its arrival, 80% of the *Staphylococcus aureus* isolated from the community would already have resistance to penicillin [100]. However, it was not until 2015 that international organizations warned about the escalation of bacterial resistance to antibiotics (AMR) and that research on therapeutic alternatives, among which bacteriophages stand out, intensified.

**Figure 6:**
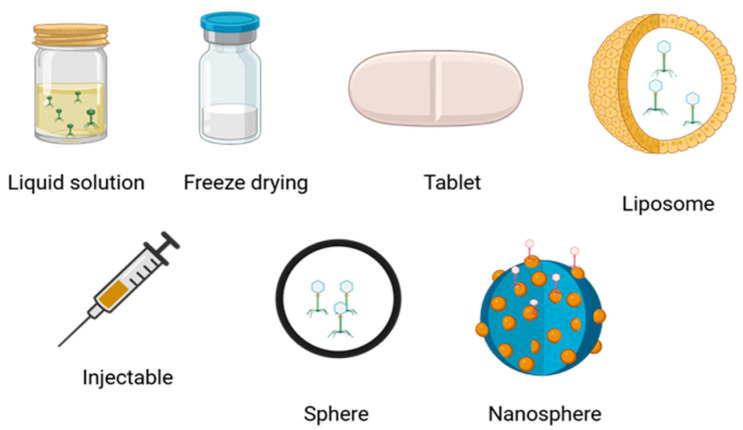
Various dosage forms for bacteriophage administration: liquid solutions, freeze drying, tablets, liposomes, injectables, spheres, and nanospheres. Created in https://BioRender.com. (https://app.biorender.com/illustrations/6738369a33ddbdfb715e45bf?slideId=177a4a8a-3c69-4577-afc0-2a888217535c, accessed on 26 December 2024).

Bacteriophages have been administered by different routes in humans, which include intravenous, pulmonary, and oral, and have targeted various multidrug-resistant bacteria, such as various varieties of *Burkholderia* spp. [101], *Escherichia coli* [102], *Klebsiella pneumoniae* [103], *Salmonella enteritidis*, *Pseudomonas aeruginosa* [104], *Acinetobacter baumanii*, *Staphylococcus aureus*, *Staphylococcus epidermidis*, *Mycobacterium tuberculosis*, among others [105]. To combat bacterial infections in skin, upper and lower respiratory tract, bones, urinary tract, cardiac, enteric, gynecological, neurological, abscesses, bacteremia, and sepsis through different delivery routes that include intravenous, intramuscular, intraosseous, rectal, otic, vaginal, vaginal, urethral, inhalational, transdermal, oral, and pulmonary (Figure 6) [106,107]. Additionally, phages have also been proposed as tools to modulate microbiomes, helping to restore microbial balance in diseases related to bacterial overgrowth [92]. For example, phages can be used to control populations of *Helicobacter pylori* or *Clostridium difficile* [108], responsible for gastric ulcers and recurrent diarrhea, respectively.

In recent years, several studies have been documented that have proven the efficacy of bacteriophages; human trials have reported efficacy percentages of 63 to 100% [109], using titers from 10^5^ to 10^11^ [110,111], and sometimes adverse effects include dizziness, headaches, and hypotension, which may disappear when decreasing the title used. However, it was reported that a two-year-old child experienced an anaphylactic reaction that is presumed to be more related to the release of bacterial endotoxins [112,113].

An important aspect to consider in phage therapy is the stability of the phages. Some phages are formulated in an aqueous solution due to the sensitivity of the proteins that encapsulate their genetic material. Additionally, a patient’s immune response may generate antibodies against bacteriophages, which can hinder their entry into target cells. However, there are formulations that use freeze-dried bacteriophages contained in spheres or nanoparticles (Figure 6). Furthermore, the stability of certain bacteriophages in solution can last up to one year [113].

Figure 6 show some bacteriophage formulations, for example, to promote the entry of phages into the area of infection, and strategies used, such as adding polymers to the formulations that can stabilize and improve them. For example, sucrose and trehalose are good cryoprotectants that reduce the loss of phage titers during the freeze-drying process [114,115,116,117]. On the other hand, polymers such as alginate, calcium, chitosan, polyphenolic extracts, agarose, pectin, hyaluronic acid methacrylate, hydroxypropyl methylcellulose, polyester amide, polyvinyl, polyethylene oxide, polyvinyl alcohol, and polymethyl methacrylate are excipients to formulate bacteriophages. Finally, the production of gold nanoparticles, aluminum oxide silica, has allowed bacteriophages to be stabilized in a controlled and effective manner in bacterial biofilms [117,118,119,120].

However, even scientists still manage to protect, encapsulate, or stabilize bacteriophages. Another limiting factor is the size of the phages. Phages are millions of times larger than antibiotics. This means their ability to cross the many barriers present in the body, from the intestinal wall to the blood–brain barrier, is limited.

Although bacteriophage therapy has shown promising results in both animals and humans [121,122], it is essential to emphasize the importance of a deep characterization of bacteriophages in terms of the absence of lysogeny genes, the use of predictive dose models, and the route and time of administration since adverse effects, resistance of bacteria to bacteriophages, or ineffectiveness of the treatment have sometimes been described, as in the case of the study by Aslam et al. [123], who report five cases of ineffectiveness in the treatment of *Pseudomonas aureginosa* infections, the same cases that had different doses and treatment times. Another aspect to consider is the importance of national and international collaboration in phage banks since time is short, and the effectiveness of phages promotes personalized treatment. Not only national regulations but also international agreements may be needed when a strain crosses borders, as in the case of infections by rare bacteria [86], perhaps extending the level of the proposal called “The Belgian model” that has worked in Europe, which proposes phage banks available when they are needed [121].

## 5. Pharmacokinetic Aspects of Bacteriophages

The World Health Organization defines pharmacokinetics as the study of drug absorption, distribution, metabolism, and elimination. Additionally, pharmacists recognize the release of the active ingredient as a step prior to the drug’s absorption [124].

In bacteriophages, the challenges of pharmacodynamics are varied, as formulations are still being researched; however, the administration of phages has been documented intravenously, intraperitoneally, orally, topically, intranasally, and subcutaneously, with the first two being the ones that have shown the best results in terms of efficiency and phage recovery. However, the rest of the routes have been adequate for bacterial elimination [125]. Some authors mention that one of the most critical challenges of phage administration is the interaction with the non-specific immune system and the concern of the interaction of bacteriophages with other bacteria [126]. A recent study used T4-like phages and introduced them into mammalian cells, where they were internalized and accumulated in specific cellular compartments called macropinosomes (membranous undulations with ruffling effect caused by some pathogens in their eukaryotic hosts). Surprisingly, these phages did not activate inflammatory pathways that are normally triggered by foreign DNA, such as TLR9 or cGAS-STING. The T4 bacteriophage activates an AKT-dependent pathway that enhances metabolism, promotes cell survival, and reorganizes actin, facilitating the virus’s entry into the host cell; the authors conclude that T4 promoted the establishment of the bacterium in the G1 phase of circular growth and promoted metabolism, which shows once again that even if bacteriophages enter the cell, they do not cause damage, at least in lung cells analyzed. Finally, through microarray techniques and fluorescence microscopy, the bacteriophage showed the ability to inhibit the BAD (BCL2) pathway (BCL2) that regulates an apoptotic stress response pathway [127], which could promote cell survival to this stimulus [128].

In summary, to administer bacteriophages, it is necessary to take into account the location of the disease, the number of bacteria in the host, the route of administration that guarantees access to the site of infection, the immune response, and the dose of phages administered, so it is still necessary to continue investigating to establish the correct calculation of the doses to patients as well as the risk of phage therapy use in immunocompromised and pregnant patients [129,130].

## 6. Pharmacodynamic Aspects of Bacteriophages

The study of pharmacodynamics considers the interaction of drugs and biological responses of administered organisms, which determines their efficiency and safety [131]. The authors describe the mechanisms of action and safety of bacteriophages. However, the two major topics to discuss in phage therapy pharmacodynamics are the quantification of bacteriophages and the calculation of the doses.

First, regarding the quantification of bacteriophage, although it has been used to quantify them by techniques such as flow cytometry [132], Electron Transmission Microscopy (TEM) [133], or molecular methods such as quantitative real-time polymerase chain reaction (QPCR) or the quantification of fluorescence emitted by a specific sequence of bacteriophages (NanoSigth) [134], the reality is that the quantification proposed by Adam in 1959, double plate, remains the most-used technique due to its cost and effectiveness. Second, the calculation of the doses used still needs to be standardized among the investigators since some only report the dose administered in their studies, and other authors have proposed mathematical models to predict the ideal doses; authors are taking into account factors such as bacterial resistance to bacteriophages, the rate of phage entry into the bacteria, and the administered phage titer or the Infection Multiplicity (MOI) achieved after phage administration [135].

Based on the information above, the term active therapy has been used to describe the phage treatment that contains the necessary dose so that the replication process reduces the bacterial load and passive treatment to the administered dose that requires at least one replicative cycle to reach the concentration needed to achieve it. This forces researchers to consider adsorption efficiency, latent period, and burst size [136]. These data are obtained experimentally with the single-step growth curve [137]. However, the physiological state of the bacteria must also be taken into account, and it must be considered that the parameters must be dynamic since each replication cycle should decrease the concentration of bacteria capable of absorbing phages, and perhaps the physiological state of the bacteria will decrease over time due to the host response and the phage attack that the bacterial population will be suffering. Observing the progress of this part of phage therapy research will be interesting.

## 7. Regulation and Challenges of Phage Therapy

Penicillin was discovered in 1928; by 1943, it produced industrial quantities, and the cost of treatment was affordable [122]. In contrast, the efficacy of bacteriophages for precisely eliminating bacteria has been described for a little over one hundred years, and there is currently no production capable of removing bacteria of greater clinical interest. There is no regulation to make such production possible in many countries [138,139]. The above is because the biology of bacteriophages and the characteristics of their replicative cycles were initially unknown. Still, later technological advances and greater knowledge of viral biology, bacterial resistance to bacteriophages, the lack of knowledge about immune cells in phages, the lack of consensus on phage dose, and the lack of dissemination of this therapeutic alternative have forced interested researchers to proceed cautiously with the use of phage therapy.

The presence of bacteriophages in the ovary, brain, and cardiac tissue of experimental animals must be understood to ensure the safety of phage therapy. It is known that an average human lives with 10^16^ plate-forming units (PFU) [140]. Because of it, it has to be interesting to understand the effect of the genetic material of bacteriophages on the genetic, protein, and metabolic regulation of host cells and eukaryotes that coexist in the microenvironment of the sick person, as proposed by Soutourina (2013), who claims to have identified, through in silico and in vitro tests, microRNAs with the ability to interfere with the regulation of bacterial genes [138]. The role of phage microRNAs in human cells is not yet well understood.

Another factor that has played a significant role in the lack of clear and homogeneous regulations is the lack of standardized controlled clinical trials [141,142]. However, to date, 53 clinical trials are being conducted in France, Switzerland, the USA, Poland, Canada, the UK, Israel, Uzbekistan, Russia, and Italy (search for infectious disease, other terms: Bacteriophages|Card Results|ClinicalTrials.gov). Ling and colleagues (2022) also systematically reviewed studies conducted in phage therapy [142]. Although not all had the same characteristics to compare them, the authors concluded that the therapy’s response improves if the phage administration is near the affected area [143].

On the other hand, several studies report no serious adverse reactions to phage therapy, and sometimes, these disappear when the dose of administered phages is reduced.

The European Union currently recognizes bacteriophages as biological medicines, and the European Pharmacopoeia announced the addition of a new chapter on the regulation of phage therapy in 2025 [128]. In this region of the world, phage therapy is more developed for historical reasons [139]; however, in the United States, phages have been approved as Generally Recognized Safe Products (GRASS), but they are conducting phase three clinical trials, which they hope will lay the groundwork for the start of discussion on the legislation for these antibacterial agents. Despite efforts to work with phages in Latin America, there are no records of the use of phages in patients or laws regulating their use [143].

Some European countries have administered phages to patients, but only in extraordinary ways, provided that a Hospital Ethics Committee endorses its use based on the Helsinki Declaration in patients for whom there are no longer conventional antimicrobial treatments. However, it is necessary that more hospitals consider instituting the use of bacteriophages within their ethics committees in clinical practice and that there are more phage banks such as those proposed by Belgium [140].

“The Belgian model” is the design used by this country to promote humanitarian therapy for patients with few opportunities for treatment and consist of having phages banks to respond fast to necessities of population [139].

It is fair to say that in terms of regulation, the world is not prepared for the emergence of resistance to antibiotics. However, many countries are working on that, but more progress is still needed, so it is essential to add efforts to promote knowledge of phage therapy, improve bacteriophage formulations, establish methodologies that will enhance dose calculation, standardize controlled clinical trials, establish interactions of bacteriophages with the immune system in both healthy and immunocompromised individuals, and provide tools for the right decision makers to make on the basis of clear scientific evidence.

## 8. Conclusions

In recent decades, the resurgence of interest in bacteriophages as a promising alternative to the growing threat of multidrug-resistant bacteria has opened new possibilities for antimicrobial therapy. Despite significant advances in understanding phage biology, their infection mechanisms, and their capacity to modify bacterial genomes, important challenges remain for their clinical implementation. Inherent limitations such as phage specificity, stability, and interactions with the immune system require a multidisciplinary approach to develop stable and effective formulations. Synthetic biology tools, such as CRISPR-based genome editing, have expanded the potential of genetically modified bacteriophages, allowing for greater precision in eradicating bacterial infections. However, the lack of standardization in clinical trials and global regulatory barriers limit their widespread adoption. As the antimicrobial resistance crisis escalates, it is crucial to research further and develop phage-based therapeutic approaches, optimizing their use for future clinical applications.

## Data Availability

No new data were created or analyzed in this study. The exchange of data is not applicable to this article.

## Supplementary Materials

The following supporting information can be downloaded at: https://www.mdpi.com/article/10.3390/microorganisms13010100/s1, Table S1: Anexo.
