# Supplementary material for: Bacteriophages: A Challenge for Antimicrobial Therapy
Source: Microorganisms. 2025 Jan 7;13(1):100. doi: 10.3390/microorganisms13010100 (PMC11767365; doi:10.3390/microorganisms13010100)
Supplement: Supplementary file 1 [file microorganisms-13-00100-s001.zip › microorganisms-3322076-supplementary.pdf]

**Table S1.** Anexo.

| Structure | | |
|---|---|---|
| **Shape** | **Family** | **Structure** |
| Tailed | *Myoviridae* | 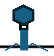 |
| | *Siphoviridae* | 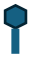 |
| | *Podoviridae* | 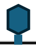 |
| Polyhedral | *Microviridae* | 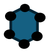 |
| | *Corticoviridae* | 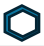 |
| | *Tectiviridae* | 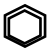 |
| | *Leviviridae* | 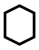 |
| | *Cystoviridae* | 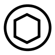 |
| Filamentous | *Inoviridae* | 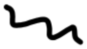 |
| | *Lipothrixviridae* | 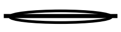 |
| | *Rudiviridae* | 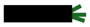 |
| Pleomorphic | *Plasmaviridae* | 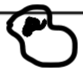 |
| | *Fuselloviridae* | 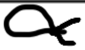 |
